# Transcriptome profiling of Nudix hydrolase gene deletions in the thermoacidophilic archaeon *Sulfolobus acidocaldarius*

**DOI:** 10.3389/fmicb.2023.1197877

**Published:** 2023-06-15

**Authors:** Ruth Breuer, José Vicente Gomes-Filho, Jing Yuan, Lennart Randau

**Affiliations:** ^1^Prokaryotic RNA Biology, Department of Biology, Philipps-Universität Marburg, Marburg, Germany; ^2^Max Planck Institute for Terrestrial Microbiology, Marburg, Germany; ^3^SYNMIKRO, Center for Synthetic Microbiology, Marburg, Germany

**Keywords:** Nudix hydrolase, transcriptomics, thermophile, RNA processing, gene regulation

## Abstract

Nudix hydrolases comprise a large and ubiquitous protein superfamily that catalyzes the hydrolysis of a nucleoside diphosphate linked to another moiety X (Nudix). *Sulfolobus acidocaldarius* possesses four Nudix domain-containing proteins (SACI_RS00730/Saci_0153, SACI_RS02625/Saci_0550, SACI_RS00060/Saci_0013/Saci_NudT5, and SACI_RS00575/Saci_0121). Deletion strains were generated for the four individual Nudix genes and for both Nudix genes annotated to encode ADP-ribose pyrophosphatases (*SACI_RS00730, SACI_RS00060*) and did not reveal a distinct phenotype compared to the wild-type strain under standard growth conditions, nutrient stress or heat stress conditions. We employed RNA-seq to establish the transcriptome profiles of the Nudix deletion strains, revealing a large number of differentially regulated genes, most notably in the Δ*SACI_RS00730/SACI_RS00060* double knock-out strain and the Δ*SACI_RS00575* single deletion strain. The absence of Nudix hydrolases is suggested to impact transcription *via* differentially regulated transcriptional regulators. We observed downregulation of the lysine biosynthesis and the archaellum formation iModulons in stationary phase cells, as well as upregulation of two genes involved in the *de novo* NAD^+^ biosynthesis pathway. Furthermore, the deletion strains exhibited upregulation of two thermosome subunits (α, β) and the toxin-antitoxin system VapBC, which are implicated in the archaeal heat shock response. These results uncover a defined set of pathways that involve archaeal Nudix protein activities and assist in their functional characterization.

## 1. Introduction

**Figure 1:**
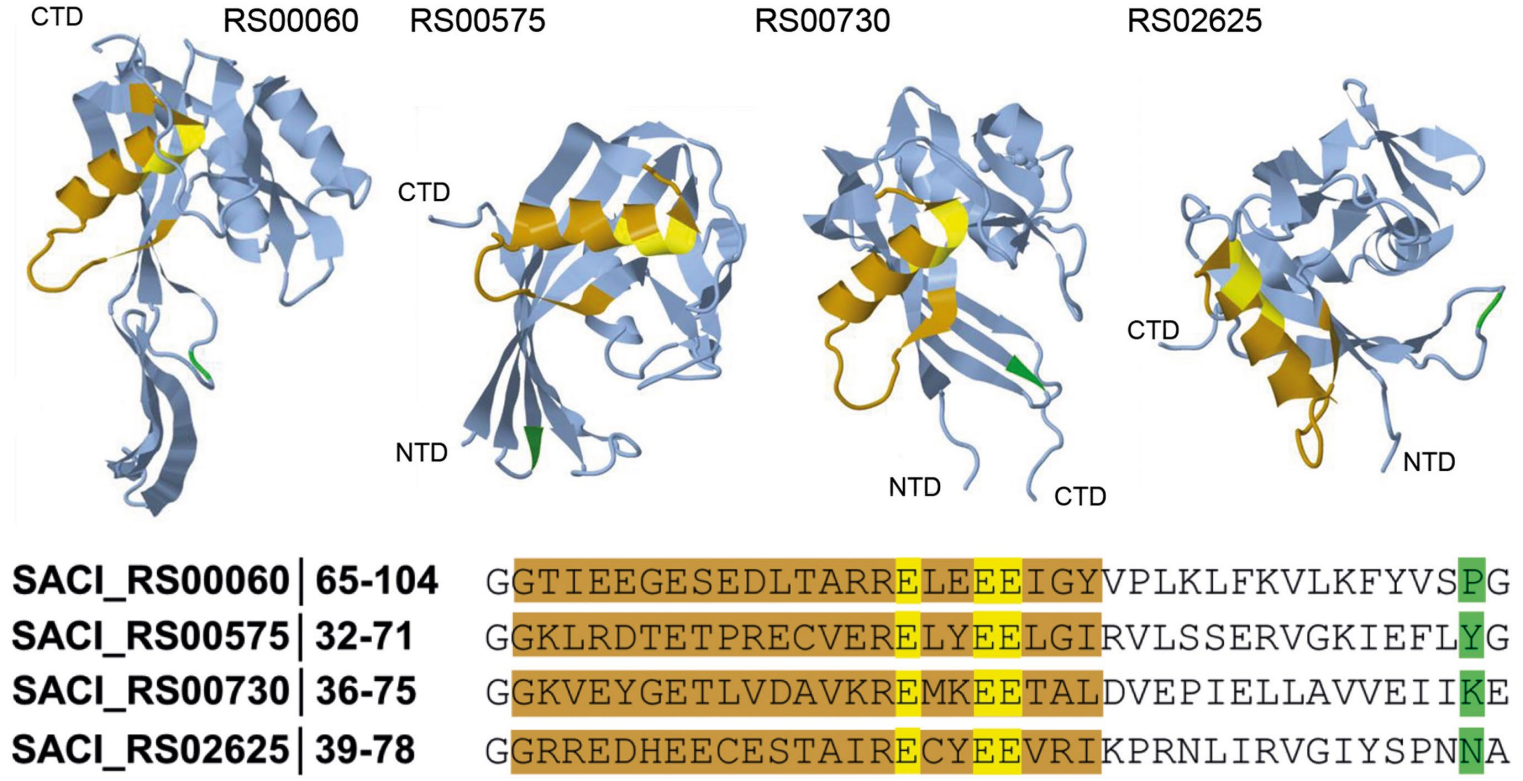
Schematic representation of the substrate backbone for Nudix hydrolases. In *S. acidocaldarius*, four Nudix hydrolases were identified, and their tridimensional structure was predicted using AlphaFold (AlphaFoldDB accession codes: Q4JCN6, Q4JCD4, Q4JCA5, Q4JB83, Jumper et al., 2021). The Nudix motif (orange, consensus sequence: GX_5_EX_7_REUXEEXGU) and the corresponding functional amino acids (yellow) are shown. The amino acid at position 16 (green) after the Nudix motif can be used as an initial guide for substrate identification of newly discovered Nudix hydrolases.

Nudix hydrolases constitute an evolutionary conserved protein superfamily of functionally versatile proteins present in all three domains of life. They catalyze the hydrolysis of a wide range of small nucleotide substrates composed of a nucleoside diphosphate linked to another moiety X (Nudix) (Figure 1) identifiable by the conserved Nudix motif with the consensus sequence GX_5_EX_5_U/AXREX_2_EEXGU (U for a hydrophobic residue, X for any residue) (Bessman et al., 1996; McLennan, 2006). Initially characterized as “housecleaning enzymes” which cleanse the cell of potentially toxic metabolites (Bessman et al., 1996), it has since been revealed that their biological roles are more diverse than previously thought. In *Escherichia coli*, the Nudix hydrolase NudB hydrolyses 8-oxo-dADP, 8-oxo-dGDP and 2-oxo-dADP and was thus proposed to possess antimutator activity (Hori et al., 2005). Subsequent studies revealed dihydroneopterin triphosphate (DHNTP), which is structurally similar to GTP, to be the preferred substrate and deletion of *nudB* led to impaired folate synthesis *in vivo*, where DHNTP plays an integral intermediary role (Gabelli et al., 2007). Recently, Nudix hydrolases have gained attention due to their ability to remove non-canonical metabolite caps from RNA molecules. Two prominent examples are the bacterial Nudix hydrolases NudC and RppH. Initially described as a NAD/H pyrophosphohydrolase, NAD-RNA decapping by NudC results in 5′-monosphosphorylated RNA and nicotinamide mononucleotide (NMN) (Frick and Bessman, 1995; Cahová et al., 2015). The RNA pyrophosphohydrolase RppH converts 5′-triphosphorylated RNA into 5′-monophosphorylated RNA triggering RNA degradation by RNase E or RNase J (Deana et al., 2008; Richards et al., 2011) and can also act as NAD-decapping enzyme (Frindert et al., 2018; Grudzien-Nogalska et al., 2019). Furthermore, *E. coli* RppH removes non-methylated 5′-Np_n_N-caps from RNA (Hudeček et al., 2020). In eukaryotes, various Nudix hydrolases (termed “NudT”) capable of removing different caps from RNA *in vitro* have been identified (Abdelraheim et al., 2003; Song et al., 2010, 2013; Grudzien-Nogalska et al., 2019). NAD-capped RNA has been identified in bacteria and eukaryotes and recently also in the archaeal domain in *Sulfolobus acidocaldarius* and *Haloferax volcanii* (Chen et al., 2009; Cahová et al., 2015; Jiao et al., 2017; Walters et al., 2017; Ruiz-Larrabeiti et al., 2021; Gomes-Filho et al., 2022). Furthermore, methylated and non-methylated dinucleoside polyphosphates (Np_n_Ns) were identified at the 5′ ends of *E. coli* RNA (Hudeček et al., 2020) and most recently, ADPR-capped RNA was identified in human cells (Weixler et al., 2022).

Following the discovery of NAD-capped RNA in *S. acidocaldarius*, we aimed to identify the respective decapping enzyme(s) among the Nudix hydrolases present in this organism and identified four Nudix domain-containing proteins encoded in the genome. *S. acidocaldarius* is a thermophilic crenarchaeon with an optimal growth temperature between 75 to 80°C and the need for acidic growth medium with an optimal pH of 2–3. It might likely possess more than one decapping enzyme, e.g., mammalian cells possess more than 20 decapping enzymes, each regulating specific subsets of capped RNA (McLennan, 2006; Song et al., 2010; Li et al., 2011). Additionally, it has been reported that the number of Nudix family representatives in bacteria, eukaryotic microorganisms and fungi is linearly correlated with genome size (McLennan, 2006). Currently, *E. coli* presents 13 genes encoding Nudix family hydrolases, *Saccharomyces cerevisiae* 4, *Caenorhabditis elegans* 14, *Drosophila melanogaster* 20 and *Arabidopsis thaliana* 25–28 members (McLennan, 2006; Bessman, 2019). In the archaeal domain, *Haloferax volcanii* exhibits 12 genes encoding Nudix domain-containing proteins, while *Methanococcous jannaschii*, *M. maripaludis* and *Thermococcus kodakarensis* each possess a single Nudix domain protein. Among the other Sulfolobales/Saccharolobales, *Sa. solfataricus* possesses 3, *S. tokodaii* 4 and *S. islandicus* 4 genes encoding Nudix domain-containing proteins. In the organisms presenting only one Nudix protein, it is annotated as a putative ADPR hydrolase (Alm et al., 2005). The Nudix family is known for displaying a large substrate range, especially *in vitro*, encompassing canonical and oxidized nucleotides, nucleotide sugars, dinucleotide coenzymes, diadenosine polyphosphates and capped RNAs, as well as non-nucleotide substrates such as inositol pyrophosphates (McLennan, 2013; Srouji et al., 2017). While the Nudix protein Saci_NudT5 demonstrated ADPR-RNA decapping ability *in vitro*, the other proteins’ activities remain elusive (Gomes-Filho et al., 2022). To this end, this study presents the impact of Nudix hydrolase absence on the transcriptome of *S. acidocaldarius*. None of the gene deletions elicited an obvious phenotype, but all strains exhibit a considerable number of differentially regulated transcriptional regulators. Overall, the transcriptome of the Nudix deletion strains resembles that of samples taken under heat stress and nutrient limitation conditions (Bischof et al., 2019), hence aiding our understanding of the gene network regulating stress response in *S. acidocaldarius*.

## 2. Results and discussion

### 2.1. The *Sulfolobus acidocaldarius* genome encodes four Nudix family hydrolases

BLAST analyses and multiple sequence alignments revealed four genes encoding Nudix domain-containing proteins in the genome of *Sulfolobus acidocaldarius*: SACI_RS00730, SACI_RS00060, SACI_RS02625 and SACI_RS00575 (Figure 1). All proteins possess the conserved glutamic acid residues in the Nudix motif crucial to Nudix activity (Cahová et al., 2015; Höfer et al., 2016; Frindert et al., 2018; Grudzien-Nogalska et al., 2019). The residue at position 16 following the G of the Nudix motif correlates with possible substrates for the respective Nudix protein and can serve to identify and distinguish different subsets of Nudix hydrolases (Dunn et al., 1999). In SACI_RS00060, a proline at this position suggests ADP-ribose (ADPR) hydrolysis activity. Indeed, this protein was recently shown to decap ADPR-RNA *in vitro* and hence renamed Saci_NudT5 to match the nomenclature of its human homolog (Gomes-Filho et al., 2022). In SACI_RS00575, a tyrosine at position 16 hints at specificity for dinucleoside polyphosphate substrates, however its substrate specificity remains unclear. Due to high sequence similarity to Saci_NudT5, SACI_RS00730 might represent another ADPR hydrolase. SACI_RS02625 is conserved across many archaeal species, but no specific activity has been predicted thus far. In bacteria, a family of Nudix-related transcriptional regulators (NrtR) regulates NAD^+^ metabolism and interacts with ADPR as their effector molecule. These proteins are characterized by an N-terminal Nudix-like domain homologous to ADPR pyrophosphatases and a C-terminal helix-turn-helix (HTH)-like DNA-binding domain (Rodionov et al., 2008). On this note, an HTH-domain was not identified in any of the Nudix family hydrolases from *S. acidocaldarius*.

Protein structures of the four Nudix hydrolases from *S. acidocaldarius* were modeled by AlphaFold sourced from the UniProt database (Jumper et al., 2021; Varadi et al., 2022; Figure 1). All protein structure predictions reveal similar folding in which the Nudix motif is present as an α-helix located on the outer part of the structure, close to the substrate pocket. In the active site, three conserved glutamic acid residues act as ligands to magnesium ions and are directed toward the inside of the pocket. The indicator residue at position 16 after the G of the Nudix motif is located on the opposite end of the pocket. Saci_NudT5, the largest of the four proteins, additionally exhibits an extended structure which is not present in the other three proteins (Figure 1).

### 2.2. The Nudix hydrolase genes are not essential in *Sulfolobus acidocaldarius*

**Figure 2:**
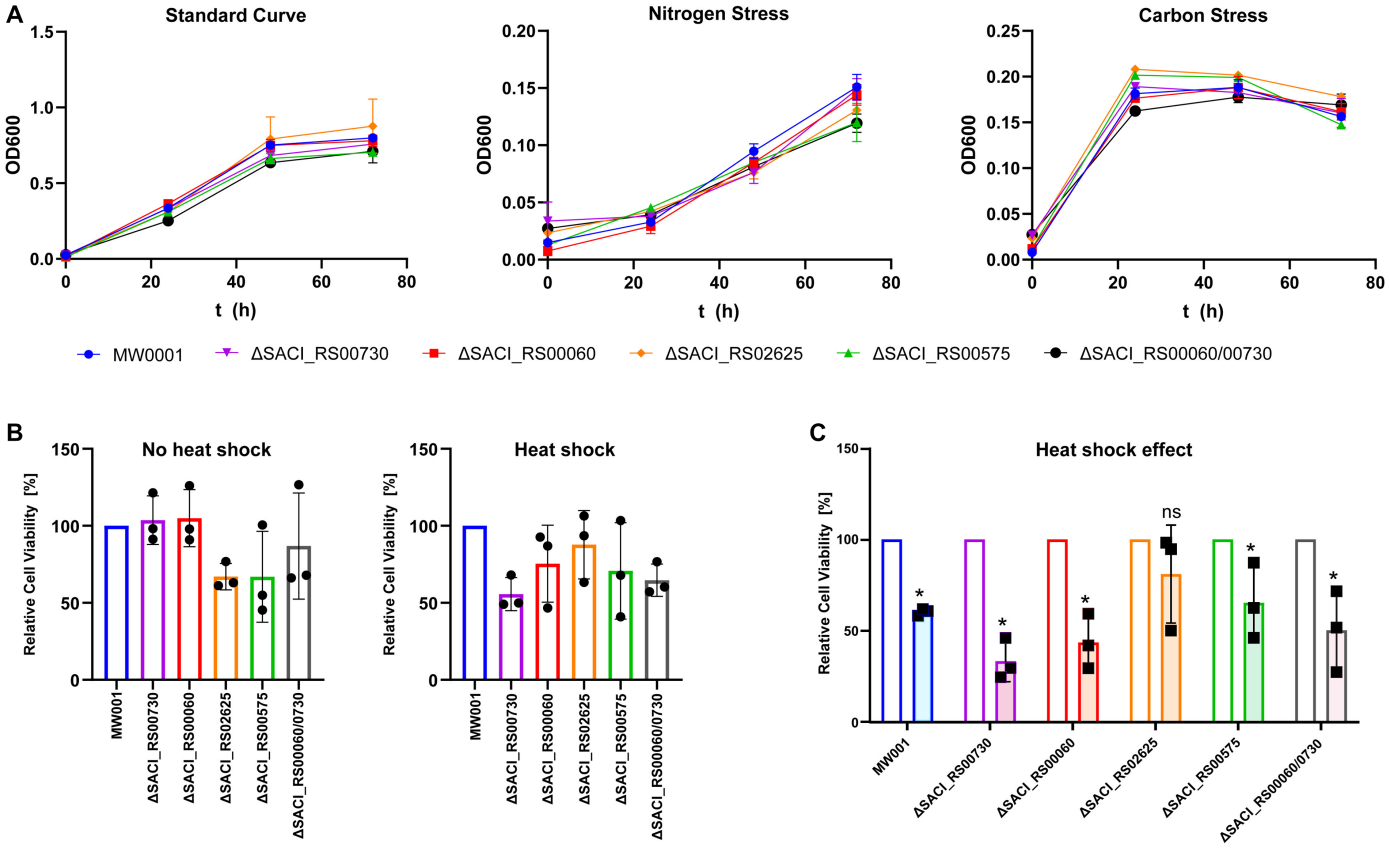
Phenotypical characterization of *S. acidocaldarius* Nudix gene deletion strains under standard and stress conditions. **(A)** Growth curves performed under standard conditions, nitrogen, and carbon stress. **(B)** Cell viability of each knockout strain relative to the wild-type with and without heat stress. **(C)** The relative cell viability after heat treatment was normalized by cell viability of non-heat-treated control samples. Each experiment was performed with triplicates, and error bars depict their standard deviation. Asterisks (*) denote Student’s *t*-test value <0.05.

The genes encoding all four Nudix hydrolases in *S. acidocaldarius*, *SACI_RS00730*, *SACI_RS00060* (encodes Saci_NudT5), *SACI_RS02625* and *SACI_RS00575* were individually targeted for deletion using the double crossover method based on plasmid pSVA431 developed by Wagner et al., 2012. With this approach, all four genes were removed from the genome without interrupting their partially overlapping neighboring genes, indicating that none of the Nudix hydrolases is essential for *S. acidocaldarius*. The absence of Nudix gene transcripts was confirmed by RNA-sequencing (Supplementary Figure S1). To account for a possible redundancy between the two most similar genes (Supplementary Figure S2), a double deletion mutant of *SACI_RS00730* and *SACI_RS00060* was generated. In a phenotypical survey, the deletion strains were grown in parallel to the wild-type strain under standard, nitrogen stress and carbon stress conditions. All strains showed similar growth behavior and no significant deviation from wild-type growth was detected (Figure 2A). Similarly, no significant deviation was observed between the wild-type and deletion strains after submission to 87°C heat shock and cell growth was significantly reduced in all strains except for Δ*SACI_RS02625* (Figure 2B). Here, heat-shocked cells showed survival rates between 33 and 65% compared to their control samples, while for ΔS*ACI_RS02625* 80% of cells were still viable after heat shock exposure (Figure 2C). In conclusion, the Nudix hydrolase genes are not essential in *S. acidocaldarius* and their deletion did not elicit a distinct phenotype compared to the wild-type strain under conditions tested.

### 2.3. Each deletion strain exhibits a distinct transcriptome profile

**Figure 3:**
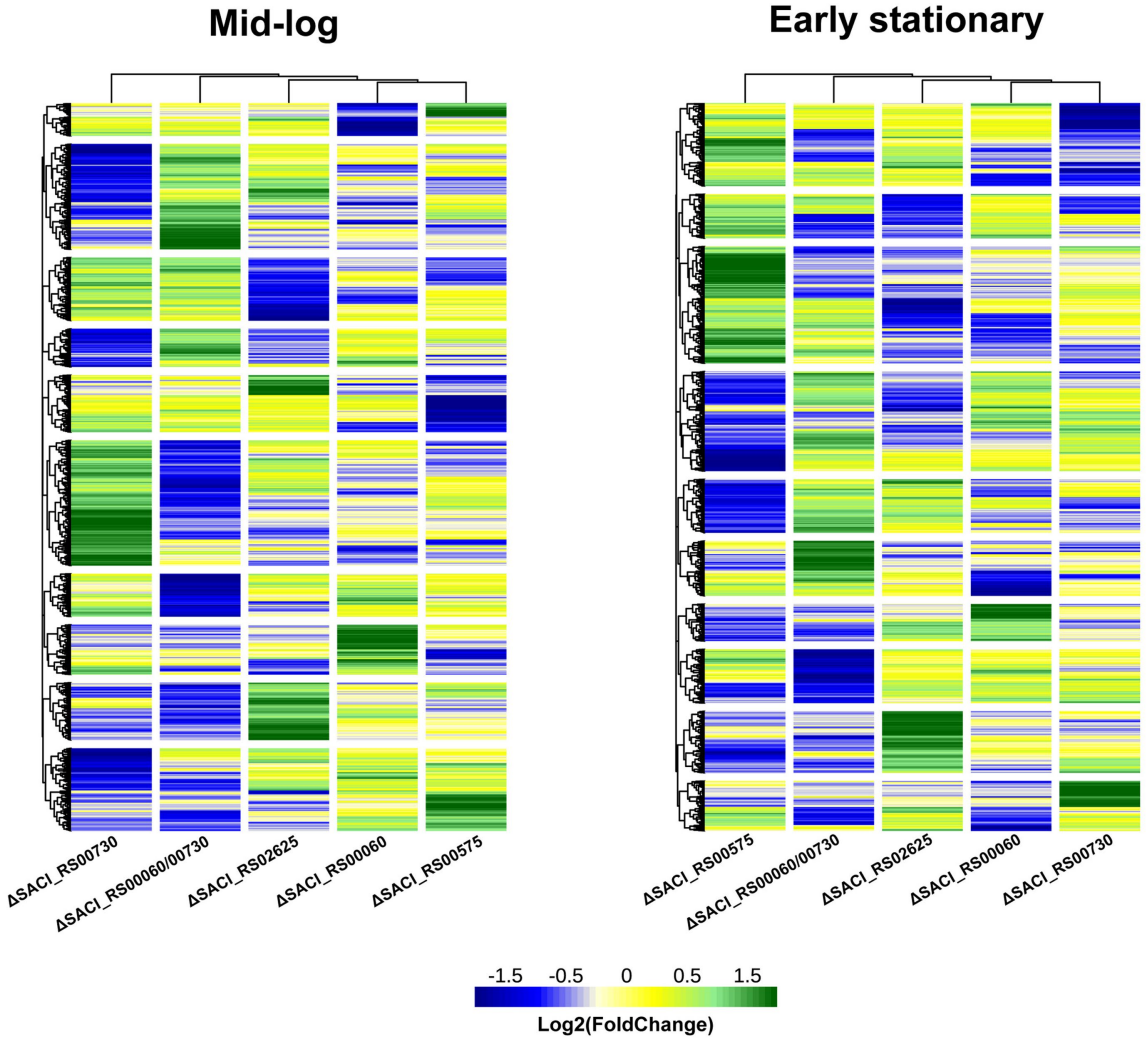
Transcriptomes of Nudix gene deletions. Heatmap of the log2 (FoldChange) of the Nudix gene deletion strains’ transcriptome relative to the wild-type strain *S. acidocaldarius* MW001 in the mid-log and early stationary growth phases and clustered according to Euclidean distances. Individual datasets can be found in Supplementary Table S1. Green: upregulated genes, blue: downregulated genes, yellow: unaffected.

The Nudix deletion strains were grown in parallel to the wild-type strain and their transcriptome profiles were determined for the mid-logarithmic and the early stationary phase using Illumina RNA-seq. Differential gene expression was analyzed using DEseq2 to enable comparison of the strains’ transcriptome profiles (Supplementary Table S1). RT-qPCR was used to independently assess the quality of RNA-seq data and verified gene expression profiles of selected genes (Supplementary Figure S3). Overall, the datasets exhibit more differentially regulated genes in the early stationary compared to the mid-log phase. Both heatmaps show a similar clustering of the strains according to the similarity of their transcriptome profiles with placement of Δ*SACI_RS00730* and Δ*SACI_RS00575* on opposite ends of the neighborhood tree, clearly establishing these two transcriptomic profiles as most dissimilar (Figure 3). The highest number of differentially regulated genes is present in the Δ*SACI_RS00730/SACI_RS00060* double deletion strain and the Δ*SACI_RS00575* strain, hence establishing them to be most impactful on the transcriptome. The smallest transcriptomic impact is caused by the individual deletions of *SACI_RS00730* and *SACI_RS00060* (Figure 3) which were initially believed to possess redundant activities due to high sequence similarity. However, the presence of oppositely regulated gene clusters, as well as distinct activities in the *in vitro* decapping assays refutes this assumption (Gomes-Filho et al., 2022). In summary, clusters of similarly affected genes are rarely shared between more than two strains, giving each strain a unique transcriptomic profile and suggesting unique roles for the enzymes in question. This agrees with the occurrence of only four Nudix hydrolase genes in the genome, as the likelihood of redundancies would be expected to increase with the number of Nudix genes.

**Figure 4:**
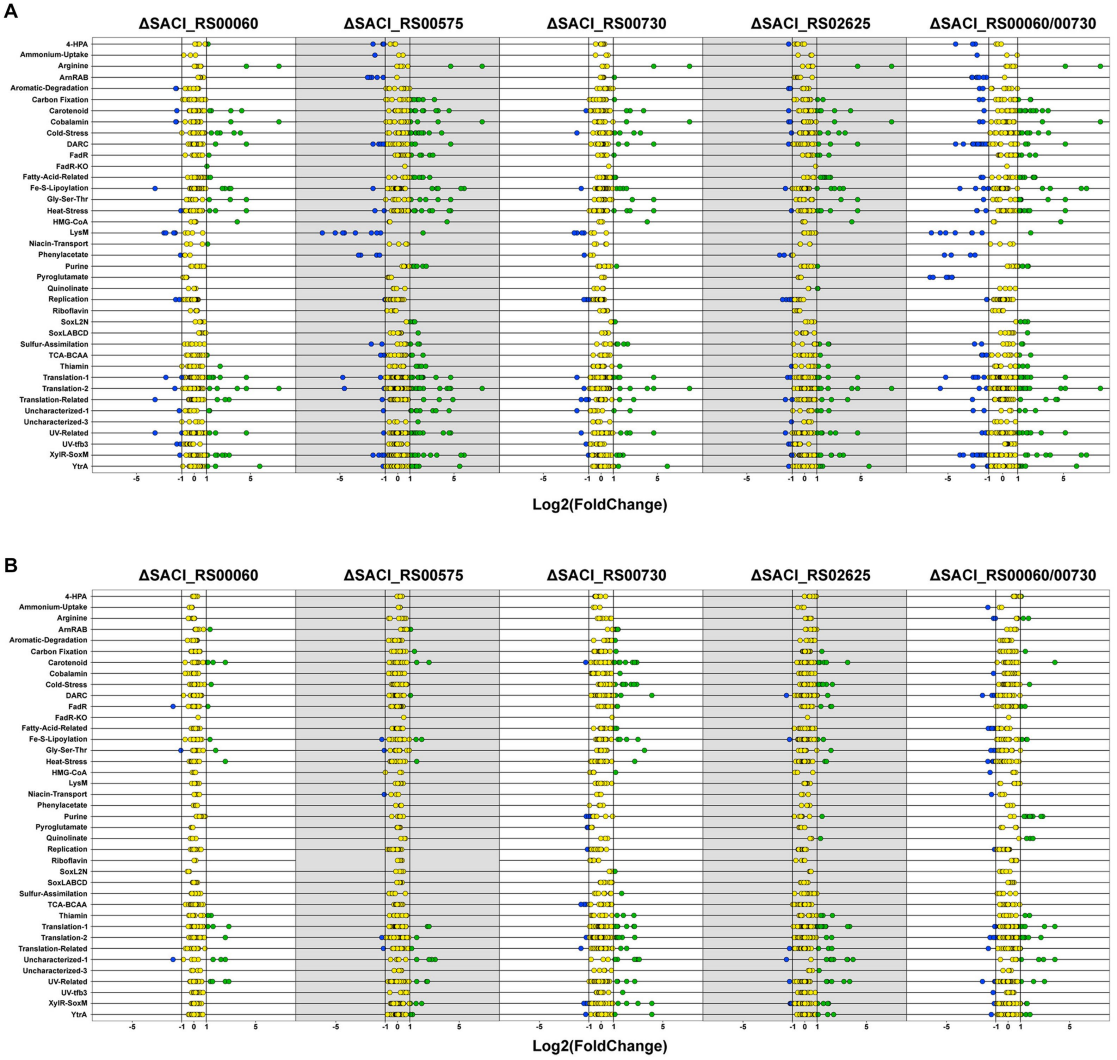
Differentially regulated genes in Nudix gene deletion strains. Dot plot of differentially regulated genes for all Nudix knockout strains in the early stationary **(A)** and mid-log phase **(B)**. Genes were assembled into clusters of similarly regulated genes based on the iModulonDB database (Chauhan et al., 2021; Rychel et al., 2021). Green: upregulated genes, blue: downregulated genes, yellow: unaffected.

To gain insight into the impact of the individual Nudix deletions, the differentially regulated genes of each dataset were assembled into iModulons according to the iModulonDB database (Chauhan et al., 2021; Rychel et al., 2021; Figure 4). An iModulon (independently modulated signal) comprises a group of genes similarly expressed under different (growth) conditions and is hence proposed to be the data-driven analog of a regulon without spatial restriction. These iModulons were identified by observing patterns in transcriptome datasets using unsupervised machine learning and independent component analysis (ICA) (Rychel et al., 2021). The genes encoding the four Nudix family hydrolases are not assigned to any iModulons. Notably, genes can be assigned to more than one iModulon and each iModulon may encompass more genes than currently displayed. Presumably, the iModulons are modulated by a common regulator or related ones which must not necessarily be part of its iModulon and for many iModulons of *S. acidocaldarius* a common regulator has not yet been identified (Chauhan et al., 2021). The DARC (Discovered signal with Absent Regulatory Components) iModulon which consists predominantly of poorly characterized genes, contains two transcription factors, one of which (*SACI_RS05830*/*saci_1223*) is upregulated in all Nudix deletion strains. A relation of this iModulon to the cell membrane was proposed, however the large number of uncharacterized genes impedes further predictions (Chauhan et al., 2021).

Between the five Nudix deletion strains, 24 genes were identified to be upregulated in all strains in the early stationary phase (Supplementary Table S2). No common genes were identified for the mid-log phase nor downregulated genes in either growth phase. Roughly half of these genes encode hypothetical proteins or proteins with domains of unknown function (DUF). Furthermore, several transcriptional regulators were affected in all deletion strains. Most of these genes are attributed to the iModulons of transcriptional regulation by XylR-SoxM or YtrA, as well as cold stress response proteins (Supplementary Table S2).

### 2.4. Nudix gene deletions indirectly affect transcription *via* transcriptional regulators

The transcriptional activator XylR regulates genes involved in xylose/arabinose uptake and its respective degradation pathway in *S. acidocaldarius*, while SoxM constitutes a terminal oxidase complex (Komorowski et al., 2002; van der Kolk et al., 2020). The respective iModulon is activated under nutrient-limited conditions, suggesting that it contains genes related to cell growth and starvation (Bischof et al., 2019; Chauhan et al., 2021). A general upregulation of numerous genes responsive to nutrient limitation and environmental stress might explain the lack of a distinct phenotype when the Nudix deletion strains were grown under carbon, nitrogen or heat stress conditions (Figure 2). Indeed, deletion of the Nudix hydrolases, especially SACI_RS00730, Saci_NudT5 and SACI_RS02625, elicits a transcriptome response highly similar to environmental stressors. Accordingly, genes attributed to several of these iModulons are significantly affected in all Nudix deletion strains and found to be mostly upregulated (Figure 4).

The Nudix deletion strains exhibit several upregulated transcriptional regulators, predominantly from the MarR family and four regulators which are present in all strains. The MarR family belongs to the super-group of transcriptional regulators present in bacteria and archaea predating the divergence of the domains (Pérez-Rueda and Collado-Vides, 2001). Its members can act as repressors or activators and their targets comprise genes involved in diverse cellular processes, such as antibiotic resistance, stress response, virulence and catabolism of aromatic compounds (Perera and Grove, 2010; Contursi et al., 2013). Hence, the effect of the Nudix gene deletions is possibly transmitted *via* interconnected transcriptional regulators. Notably, the highest number of differentially regulated transcriptional regulators is found in the datasets that contain the highest number of differentially regulated genes overall. On the same note, the number of differentially regulated genes also correlates with the number of upregulated thermosome subunits and Type II toxin-antitoxin (TA) system genes: one in Δ*SACI_RS00730* and Δ*SACI_RS00060*, four in Δ*SACI_ RS00575*, and five in Δ*SACI_RS02625* and the double knock-out. In *Saccharolobus solfataricus*, the toxin VapC6 is a heat-dependent ribonuclease which is inactivated by VapB6 binding. The ribonucleolytic activity is suggested to aid in the repression of protein synthesis during heat shock response (Maezato et al., 2011). Another study in *Sa. solfataricus* found *vapBC* locus expression to be heat-induced, however some *vapBC* loci were also expressed under normal growth conditions, suggesting roles beyond heat stress response for this system (Tachdjian and Kelly, 2006). The upregulation of a putative *vapBC* locus (encoded by *SACI_RS10050/saci_2079* and *SACI_RS10055/saci_2080*) in two Nudix deletion strains corroborates this idea. Interestingly, the same study not only found a large number of MarR family transcriptional regulators upregulated in the heat-shocked *Sa. solfataricus* samples, but also upregulation of *sso_3167* which encodes a Nudix family hydrolase (Tachdjian and Kelly, 2006). The homolog of *sso_3167* in *S. acidocaldarius* is *SACI_RS02625*, whose deletion strain did not show significantly reduced growth following the 87°C heat shock (Figure 2). Another study on heat shock response in *S. acidocaldarius* revealed high upregulation of the repressor YtrA (encoded by *SACI_RS08880*/*saci_1851*) upon heat stress (Baes et al., 2020). Though YtrA itself is not affected in the Nudix deletion strains, its iModulon is predominantly upregulated in Δ*SACI_RS02625*, Δ*SACI_RS00575* and the double KO strain (Figure 4). Notably, YtrA does not regulate known heat shock proteins but two putative membrane proteins of unknown function and though its connection to thermal stress remains unclear, a recent study links transcriptional regulators such as YtrA and Type II TA systems with thermal stress response in thermophilic archaea (Lemmens et al., 2019; Cooper et al., 2023). While the exact nature of the connection between Nudix hydrolases, specifically SACI_RS02625, and the heat stress response system remains unclear, our results suggest it to be more than coincidental. Notably, a definite transcription factor regulating heat shock response in the Sulfolobales remains to be identified.

The LysM iModulon represents lysine biosynthesis in *S. acidocaldarius* and contains specifically (though not exclusively) the genes of the lysWXJK operon (Chauhan et al., 2021). This operon encodes bi-functional enzymes involved in lysine and arginine biosynthesis and is activated by LysM. In turn, LysM is inactivated by lysine and excess amounts of lysine were shown to inactivate the expression of the operon, leading to a shutdown of arginine synthesis (Brinkman et al., 2002; Ouchi et al., 2013). The LysM iModulon is downregulated in the early stationary phase in the Δ*SACI_RS00575* strain and the double KO strain (Figure 4). However, downregulation of this operon was also observed under nutrient limitation (Bischof et al., 2019). Notably, the lysine biosynthesis operon is downregulated in the double KO strain but not in the respective single deletion strains Δ*SACI_RS00730* and Δ*SACI_RS00060* (Figure 4).

In *S. acidocaldarius*, the archaellum formation operon is controlled by the inducers ArnR and ArnR1 and the repressors ArnA and ArnB and represented in the datasets by the ArnRAB iModulon (Reimann et al., 2012; Lassak et al., 2013). ArnR (*SACI_RS05625*/*saci_1180*) and the archaellum formation iModulon are downregulated in Δ*SACI_RS00575*, in contrast to upregulation of ArnR1 (*SACI_RS05580*/*saci_1171*) and the archaellum formation iModulon in Δ*SACI_RS00730* (Figure 4). The archaellum repressors ArnA and ArnB are not affected in any of the Nudix deletion strains. ArnR was observed to be induced under tryptone starvation conditions and is suggested to be controlled by a still unidentified superior transcription factor (Lassak et al., 2013). ArnR1 is upregulated in the double knock-out while the remaining archaellum formation iModulon is downregulated (Figure 4). ArnR and ArnR1 both promote motility to a different extent, as deletion mutants of *arnR* and *arnR1* exhibited a strongly and mildly diminished motility phenotype, respectively (Lassak et al., 2013). Several studies in different archaeal organisms report archaellum formation to be either repressed or stimulated under different nutrient limitation conditions (Mukhopadhyay et al., 2000; Szabó et al., 2007; Hendrickson et al., 2008; Xia et al., 2009). Arguably, intracellular nutrient limitations in Δ*SACI_RS00575* and the double knock-out strain may cause the cells to shut down energy-consuming archaellum production. Hence, downregulation of the archaellum formation iModulon may be indicative of the cell’s energy state in consequence of Nudix gene deletion(s).

Furthermore, Δ*SACI_RS02625* and the double KO strain exhibit upregulation of *nadA* (a putative quinolinate synthase) and *nadB* (a putative L-aspartate oxidase). In the genome, *nadB* is located directly upstream of *SACI_RS02625*, however its upregulation in the double KO strain refutes a locational effect. Both gene products presumably catalyze the initial two steps in the NAD^+^ *de novo* biosynthesis pathway, as inferred from homology to *T. kodakarensis*. Upregulation of the *de novo* synthesis pathway is possibly elicited by a disturbance in the NAD^+^ salvage pathway, which involves recycling NAD^+^ from nicotinamide and ADPR following its thermal degradation (Hachisuka et al., 2018). Other genes involved in these pathways were not detected to be co-regulated in the respective datasets.

### 2.5. Concluding remarks

The genome of *S. acidocaldarius* encodes four Nudix domain-containing proteins, which were shown to be not essential to the organism under conditions tested. While preliminary phenotypical screenings did not exhibit a distinct deletion phenotype compared to the wild type strain, RNA-seq revealed unique transcriptome profiles for each Nudix deletion strain. Their transcriptomes were shown to be highly affected, especially regarding iModulons which assemble genes responsive to nutrient limitation and heat stress. Considering the high number of affected transcriptional regulators, the Nudix hydrolases might be involved in the pathways of metabolites that act as effector molecules to the transcriptional regulators, leading to an altered transcriptomic state as a consequence of altered metabolite composition. Indeed, the transcriptomes of the Nudix deletion strains exhibit remarkable similarity to the transcriptome in response to nutrient limitation stress (Bischof et al., 2019). Alternatively, Nudix hydrolases could also affect transcription *via* direct regulation of the levels of (capped) transcripts. We expect that these results stimulate the characterization of the Nudix hydrolases from *S. acidocaldarius*, which is proposed to include metabolomic profiling of the Nudix deletion strains.

## 3. Materials and methods

### 3.1. Generation of Nudix deletion strains

This work uses the uracil auxotrophic strain *Sulfolobus acidocaldarius* DSM639 MW001 (Wagner et al., 2012). Cultures were grown aerobically at 120 rpm and 75°C in Brock media at pH 3.5 (Brock et al., 1972). The media was supplied with 0.1% (w/v) NZ-amine, 0.2% (w/v) dextrin and 10 μg/ml uracil. Cell growth was determined by measuring the optical density (OD) at 600 nm with a cell density meter (Amersham Biosciences). The generation of markerless Nudix deletion strains was conducted using the deletion plasmid pSVA431, as described in Wagner et al. (2012). Briefly, this plasmid carries a dual marker system consisting of the uracil cassette *pyrEF* and the *lacS* gene from *Saccharolobus solfataricus* plus two multiple cloning sites, harboring part of the gene of interest and its upstream and downstream flanking regions, respectively. The entire cassette is transformed as a linear fragment and integrated into the genome *via* homologous recombination. Plasmids were constructed using Gibson Assembly (Gibson et al., 2009) with the primers listed in Supplementary Table S3. The genes encoding all four Nudix proteins SACI_RS00730, Saci_NudT5 (SACI_RS00060), SACI_RS02625 and SACI_RS00575 were individually deleted from the genome of *S. acidocaldarius* DSM639 MW001 without interrupting their partially overlapping neighboring genes. To generate the double deletion strain, competent cells from the Saci_NudT5 deletion strain were transformed with the linear marker cassette targeting *SACI_RS00730*. The successful removal of the Nudix genes was subsequently verified by Sanger sequencing of PCR products amplified from the deletion loci and RNA-sequencing. For PCR analysis of *S. acidocaldarius* cells, 20 μl cell culture was lysed in 20 μl 0.2 M NaOH for 5 min at RT, neutralized by addition of 80 μl 0.2 M Tris–HCl pH 6.5 and 5 μl suspension was used in a 20 μl reaction using DreamTaq DNA Polymerase (Thermo Scientific). Genomic DNA of *S. acidocaldarius* was isolated from 2 mL late logarithmic-phase cultures using the NucleoSpin® Tissue Kit (Macherey-Nagel), according to the manufacturer’s instructions for cultured cells.

### 3.2. Preparation of electrocompetent *Sulfolobus acidocaldarius* cells

Cells were grown in 50 mL Brock medium supplied with 0.1% (w/v) NZ-Amine, 0.2% (w/v) dextrin and 10 μg/ml uracil, pH 3.5, at 75°C and 120 rpm to OD_600_ = 0.3–0.7. A calculated amount of culture was subsequently transferred into 50 ml fresh medium, grown to OD_600_ = 0.2–0.3 and then incubated on ice for 10–15 min. Cells were harvested by centrifugation for 15–20 min at 2500 × *g* and 4°C and the pellet was washed three times with each 30 ml of ice-cold 20 mM sucrose. Next, the pellet was resuspended in 1 ml of ice-cold 20 mM sucrose, transferred to a 1.5 ml aliquot and centrifuged for another 5 min at 2500 × *g*, 4°C. Finally, the pellet was resuspended in 20 mM ice-cold sucrose to a theoretical OD_600_ = 20 and 50 μl aliquots were stored at −80°C without the use of liquid nitrogen until further use.

### 3.3. Transformation of *Sulfolobus acidocaldarius*

Prior to transformation into *S. acidocaldarius*, all plasmids or linearized DNA fragments were methylated to circumvent the activity of the restriction endonuclease SuaI (Berkner et al., 2007). To this end, plasmids were transformed into the strain *Escherichia coli* ER1821 (New England Biolabs) carrying the plasmid pM.ESABC4I. The methylated deletion plasmids were digested with NotI-HF (New England Biolabs) to yield linear fragments and electroporated in 1 mm Gene Pulser® electroporation cuvettes (Bio-Rad) with a constant time protocol using the input parameters 1.5 kV, 25 μF and 600 Ω on a Gene Pulser® II electroporation system (Bio-Rad). Recovery was performed for 30 min at 75°C, 300 rpm, in Brock Recovery Medium (Brock medium supplied with 0.1% (w/v) NZ-Amine, no pH adjustment), before plating cells on uracil-lacking first selection plates. The plates were wrapped in wet paper towels, placed in plastic boxes to avoid drying out and incubated for 7 days at 75°C. For blue-white screening, plates were sprayed with 25 mg/mL X-gal in DMF diluted 1:5 in 20% (w/v) dextrin and incubated for 30 min at 75°C.

### 3.4. Isolation of total RNA

*Sulfolobus acidocaldarius* DSM639 and Nudix deletion strains were grown in duplicates in Brock media supplied with 0.1% (w/v) NZ-Amine, 0.2% (w/v) dextrin and 10 μg/ml uracil at 75°C, 120 rpm, to an OD_600_ = 0.3 and 0.7, corresponding to mid-log and early stationary growth phases, respectively. 2 mL culture samples were pelleted by centrifugation for 15 min at max. Speed, RT, and total RNA was isolated using the mirVana™ miRNA Isolation Kit (Invitrogen) according to the manufacturer’s instructions. Subsequently, total RNA extractions were digested with 1 U DNaseI/μg RNA (New England Biolabs) for 2 h at 37°C and cleaned up using the Monarch® RNA Cleanup Kit (50 μg) (New England Biolabs). Quantitation of RNA samples was performed using the Qubit™ RNA High Sensitivity Assay (Agilent Technologies) and Qubit® 2.0 fluorometer (Thermo Fisher Scientific GmbH).

### 3.5. Library preparation for RNA-sequencing

Ribosomal RNAs were depleted using the Pan-Archaea riboPOOL probes (siTOOLs Biotech) and streptavidin-coated magnetic beads (siTOOLs Biotech) according to the manufacturer’s instructions. Depleted RNA samples were cleaned up with the Monarch® RNA Cleanup Kit (10 μg) (New England Biolabs) and successful rRNA depletion was verified with the RNA 6000 Pico Assay for the Agilent Bioanalyzer (Agilent Technologies) according to the manufacturer’s instructions. The preparation of cDNA libraries from rRNA-depleted total RNA was performed using the NEBNext® Ultra™ II Directional RNA Library Prep Kit for Illumina® (New England Biolabs), AMPure XP SPRI beads (Beckman Coulter) and NEBNext® Multiplex Oligos for Illumina® (New England Biolabs), following the instructions of the NEBNext Library Prep Kit. Quality control and size distribution of the cDNA libraries was assessed with the High Sensitivity DNA Assay for Bioanalyzer (Agilent Technologies). Sequencing was performed as 150 nt single reads on an Illumina® NextSeq550 at the Genomics Core Facility of the Philipps University Marburg.

### 3.6. Analysis of Illumina RNA-Seq data

Raw reads were adapter and quality trimmed using Cutadapt (v2.8) and checked with FASTQC (v0.11.9) (FastQC, 2015). Processed reads (≥18 nt) were mapped to the reference genome of *S. acidocaldarius* DSM639 (NC_007181.1) using Hisat2 (v2.2.1) (Kim et al., 2019). Multiple mapped reads with the exact match score were randomly distributed. After the strand-specific screening, HTSeq (v2.0.2) was used to count gene hits (Anders et al., 2015). Statistical and differential expression analyses were performed with DESeq2 (v1.36.0) and genes with a log2 fold-change (≤ −1 or ≥ 1), value of *p* <0.05, and adjusted value of *p* <0.1 were considered differentially expressed (Love et al., 2014). Next, genes were classified according to previously established iModulons (Chauhan et al., 2021) and their general expression profiles were analyzed. The Integrative Genomics Viewer (IGV, v2.13.2) was used for data inspection (Robinson et al., 2011).

### 3.7. RT-qPCR analyses

Total RNA samples (0.04 ng/μl) were used as template for RT-qPCR analysis using the KAPA SYBR fast one-step qRT-PCR kit (Merck) following the manufacturer’s instructions. The reactions were carried out in a CFX384 Touch real-time PCR detection system (Bio-Rad). The gene SACI_RS06385 was used as an internal control. All primers used for RT-qPCR analysis are listed in the Supplementary Table S3. Each RNA sample was tested in triplicates, and data was analyzed using CFX Manager software (Bio-Rad).

### 3.8. Growth curves

*Sulfolobus acidocaldarius* DSM639 and Nudix deletion strains were grown as pre-cultures in 25 ml Brock media supplied with 0.1% (w/v) NZ-Amine, 0.2% (w/v) dextrin and 10 μg/ml uracil at 75°C, 120 rpm. Upon reaching the stationary phase, a calculated volume of each strain was transferred into 50 ml fresh medium corresponding to a starting OD_600_ = 0.01. Each strain was grown in triplicates in Brock media + NZ-Amine + Dextrin + Uracil or Brock + Dextrin + Uracil or Brock + NZ-Amine + Uracil. At the indicated time points, 200 μl from each culture were transferred into a 96 well plate and adsorption at 600 nm was measured using a CLARIOstar® *Plus* microplate reader (BMG Labtech).

### 3.9. Heat shock spotting assays

Heat shocking spotting assays were modified from Baes et al. (2020). *S. acidocaldarius* DSM639 and Nudix deletion strains were grown in triplicates in Brock media supplied with 0.1% (w/v) NZ-Amine, 0.2% (w/v) dextrin and 10 μg/ml uracil at 75°C, 120 rpm, until reaching mid-logarithmic phase. Samples were transferred into pre-warmed aliquots and incubated for 15 min at 75°C, 300 rpm, in a thermomixer (StarLab GmbH) (“adaption period”). After removing a heat shock-control sample from each tube, tubes were covered with gas-permeable sealing membrane (Breathe-Easy, Diversified Biotech) and heat shock was administered for 30 min at 87°C, 300 rpm, on the thermomixer, using a digital pocket thermometer with a K-type probe (Traceable® Products) to monitor the temperature inside the liquid. Heat shock and control samples were diluted down to OD_600_ = 0.1, followed by the preparation of a 10^−1^ to 10^−6^ dilution series in Brock Recovery medium [Brock medium supplied with 0.1% (w/v) NZ-Amine, no pH adjustment]. Finally, 3 μl of each dilution of each sample were spotted onto a solid Brock Gelrite plate supplied with 0.1% (w/v) NZ-Amine, 0.2% (w/v) dextrin and 10 μg/ml uracil and plates were incubated in a plastic box lined with wet paper towels at 75°C. After 5 days, plates were photographed and cell viability was determined by measuring spot density from the 10^−4^ dilution step using the oval selection and area measurement tools from Fiji (Schindelin et al., 2012).

## Data availability statement

The datasets presented in this study can be found in the online repository European Nucleotide Archive (ENA) under the accession number PRJEB60684.

## Author contributions

RB, JG-F, and LR designed the experiments. RB, JY, and JG-F performed the experiments. RB, JY, and JG-F analyzed the data. RB wrote the manuscript with input from JG-F and LR. All authors reviewed and edited the manuscript.

## Funding

This work was funded by the German Research Foundation (DFG) (Grant RA 2169/8–1 to LR). Open Access funding was provided by the Open Access Publishing Fund of Philipps-Universität Marburg with support of the Deutsche Forschungsgemeinschaft (DFG, German Research Foundation).

## Conflict of interest

The authors declare that the research was conducted in the absence of any commercial or financial relationships that could be construed as a potential conflict of interest.

## Publisher’s note

All claims expressed in this article are solely those of the authors and do not necessarily represent those of their affiliated organizations, or those of the publisher, the editors and the reviewers. Any product that may be evaluated in this article, or claim that may be made by its manufacturer is not guaranteed or endorsed by the publisher.
