# Supplementary material for: Transcriptome profiling of Nudix hydrolase gene deletions in the thermoacidophilic archaeon Sulfolobus acidocaldarius
Source: Front Microbiol. 2023 Jun 15;14:1197877. doi: 10.3389/fmicb.2023.1197877 (PMC10311068; doi:10.3389/fmicb.2023.1197877)
Supplement: Supplementary file 1 [file Data_Sheet_1.PDF]

Supplementary material

Transcriptome profiling of Nudix hydrolase gene deletions in the thermoacidophilic archaeon *Sulfolobus acidocaldarius*

Ruth Breuer[1], José Vicente Gomes-Filho[1], Jing Yuan[2,3] and Lennart Randau[1,3*]

[1]Prokaryotic RNA Biology, Department of Biology, Philipps-Universität Marburg, Marburg, Germany

[2]Max Planck Institute for Terrestrial Microbiology, Marburg, Germany

[3]SYNMIKRO, Center for Synthetic Microbiology, Marburg, Germany

Figure S1 - Read coverage analysis for each Nudix gene knockout strain confirms their deletion.

Figure S2 - Alignment of amino acid sequences of Saci_RS00730 and SACI_RS00060 proteins.

Figure S3 - Verification of RNA-seq results via RT-qPCR

Supplementary Table 1 – DESeq2 results containing expression profiles for all *S. acidocaldarius* genes for each knockout strain in either mid-log or early stationary growth phase.

The $\log_2$FoldChange column indicates the direction and magnitude of the differential expression (upregulated >= 1, downregulated <= -1). The p-value and padj columns measure the statistical significance of the differential expression. A gene was considered to be significantly up or downregulated if the p-value was < 0.05 and the padj < 0.1

Supplementary Table 2 – List of genes that show upregulation in all knockout strains in the early stationary phase (from Supplementary Table 1).

Supplementary Table 3 – List of plasmids and oligonucleotides used in this study.

Figure S1

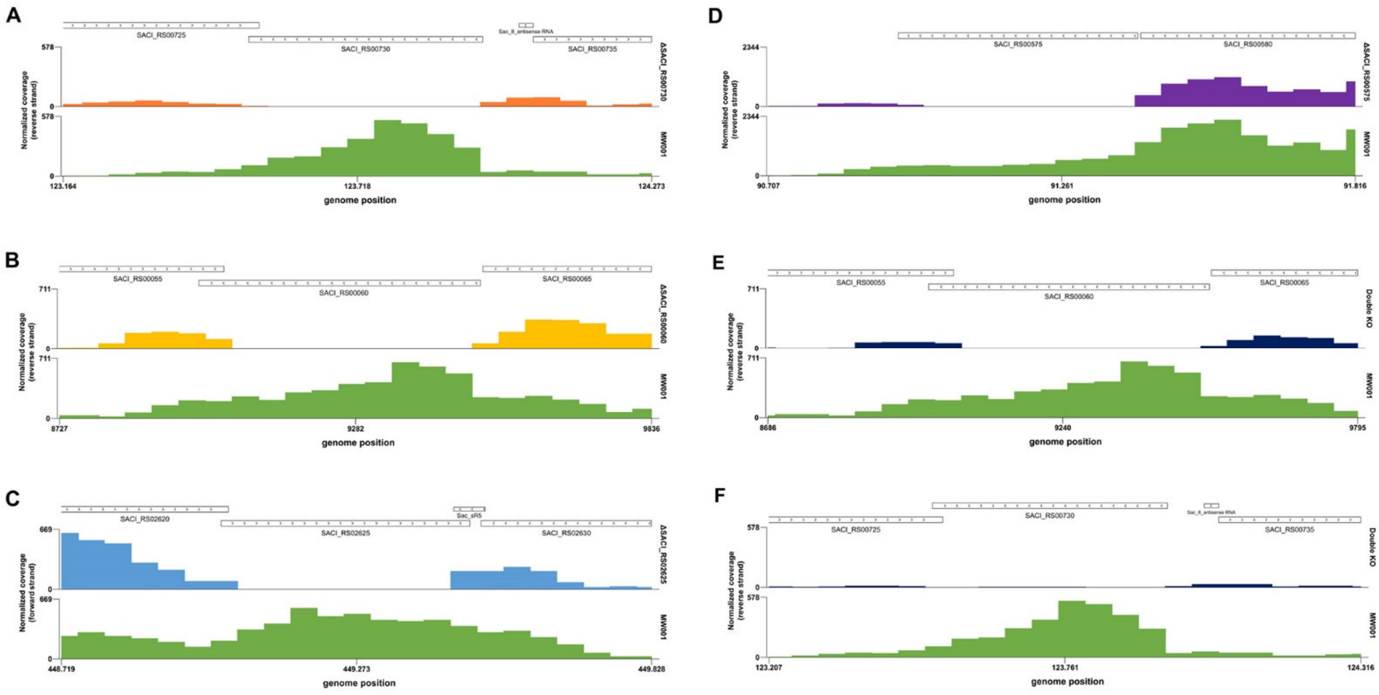

Supplementary Figure 1 – Read coverage analysis for each Nudix gene knockout strain confirms their deletion. A) ΔSACI_RS00730, B) ΔSACI_RS00060, C) ΔSACI_RS02625, D) ΔSACI_RS00575, E,F) ΔSACI_RS00730/ΔSACI_RS00060. Read coverage was normalized for sequencing depth for each strain.

Figure S2

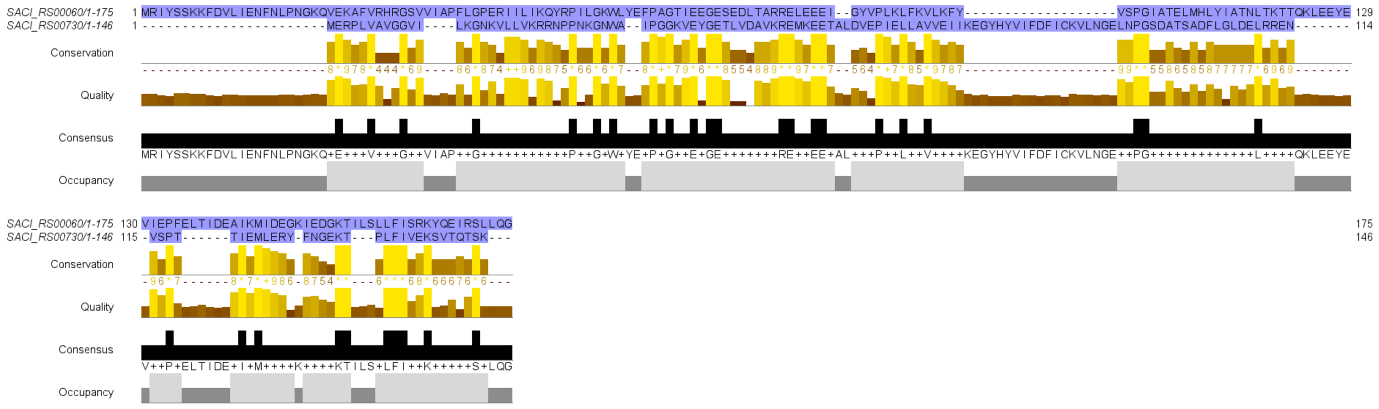

Supplementary Figure 2 – Alignment (ClustalW2) of amino acid sequences of Saci_RS00730 and SACI_RS00060 proteins.

Figure S3

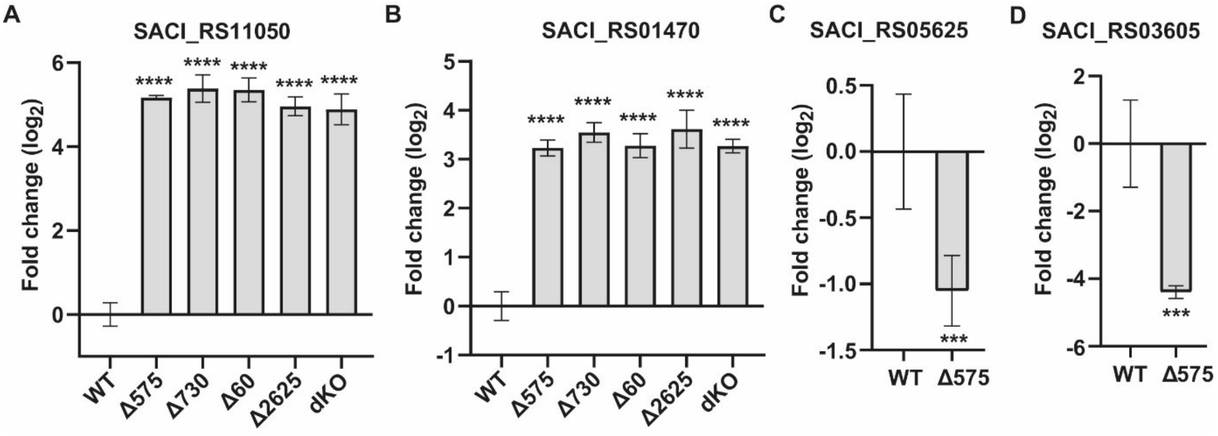

Supplementary Figure 3 - Verification of RNA-seq results via RT-qPCR. Relative changes at the mRNA level were confirmed by RT-qPCR using primers (Supplementary Table S3) within the coding regions of respective genes. The transcription of genes SACI_RS11050 (A) and SACI_RS01470 (B) increased significantly in the indicated mutant strains compared to the wild type ($P < 0.0001$, t-test). SACI_RS05625 (C) and SACI_RS03605 (D) transcripts showed significant downregulation in the Δ575 strain ($P < 0.001$). All data points represent averages of two biological and three technical replicates, with error bars indicating standard deviations.
